# Data highlighting the effects of spinal segmental stimulation of preganglionic sympathetic neurons on the electrophysiology of the rabbit heart

**DOI:** 10.1016/j.dib.2018.04.077

**Published:** 2018-04-24

**Authors:** Reshma A. Chauhan, John Coote, Emily Allen, Pott Pongpaopattanakul, Kieran E. Brack, G. Andre Ng

**Affiliations:** a Department of Cardiovascular Sciences, University of Leicester, UK; b University of Birmingham, UK; c NIHR Leicester Cardiovascular Biomedical Research Centre, Leicester, UK; d University Hospitals of Leicester NHS Trust, Leicester, UK

## Abstract

This article presents data highlighting the functional selectivity of cardiac preganglionic sympathetic neurons in the rabbit heart. Specifically, the data draw attention to the role of each spinal segmental outflow on cardiac electrophysiology and the influence of each segment on cardiac excitability through investigating markers of arrhythmia such as electrical restitution. This data holds importance for exploring whether the preganglionic sympathetic neurons have functionally distinct pathways to the heart and whether some spinal segmental outflows have a greater potential for arrhythmia generation than others. Discussion of the data can be found in Chauhan et al. (2018) [1].

**Specifications Table**

**Table:** 

Subject area	*Cardiovascular Sciences*
More specific subject area	*Cardiac electrophysiology*
Type of data	*Figures*
How data was acquired	*Functional and electrophysiological recordings were obtained from the innervated isolated rabbit heart preparation using pressure transducers (MLT0380/D ADInstruments Ltd, Charlgrove, UK), monophasic action potential electrodes (Harvard Apparatus Ltd, Holliston, Massachusetts, US. Model number 73–0150) and platinum hook electrodes (Grass Instruments, Astro-Med Inc., USA). The Powerlab 16/30 system (AD Instruments Ltd, Chalgrove, UK) was used to record the signals.*
Data format	*Raw and analyzed*
Experimental features	*The right and left sympathetic chains were isolated and stimulated at different spinal segmental levels using custom made electrodes.*
Data source location	*Leicester, United Kingdom*
Data accessibility	*Data is available with this article*

**Value of the data**

• This data improves the understanding of the differential effects of right and left sympathetic chain stimulation on cardiac electrophysiology.

• This data highlights the different responses elicited when stimulating the right and left sympathetic chains at different spinal segmental levels.
• The present dataset is valuable for identifying spinal segments of the right or left sympathetic chains displaying a dominant effect on cardiac electrophysiology, which could have implications in refining treatment modalities involving sympathetic innervation such as stellectomy.

## 1 Data

### 1.1 Chronotropic and inotropic effects

**Fig. 1:**
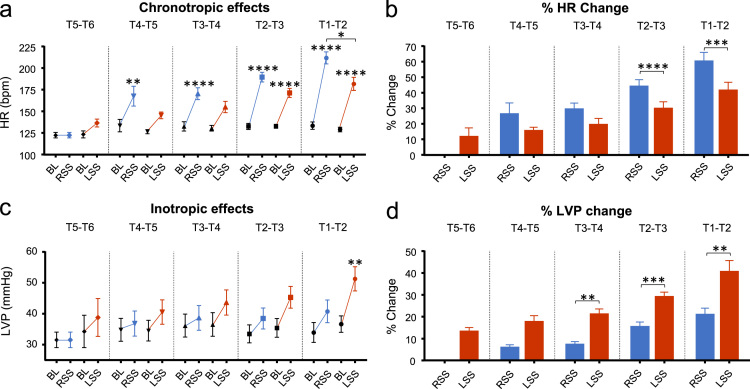
The effect on heart rate (HR) and left ventricular pressure (LVP) with right and left sympathetic stimulation at different spinal segments. The effect on heart rate (HR) and left ventricular pressure (LVP) with right and left sympathetic stimulation at different spinal segments. (a) Intrinsic HR response from baseline (BL) to right sympathetic stimulation (RSS) and BL to left sympathetic stimulation (LSS) at spinal segments T1–T6. (b) Percentage HR change for RSS and LSS at spinal segments T1–T6. (c) LVP response during constant ventricular pacing (250 bpm) from BL to RSS and BL to LSS at spinal segments T1–T6. (d) Percentage LVP change for RSS and LSS at spinal segments T1–T6. Data represent mean ± SEM. *P< 0.05, **P< 0.01, ***P< 0.001, ****P< 0.0001.

At T5–T6, stimulation of the left sympathetic chain increased HR to 136.5 ± 4.6 bpm (11.2 ± 6.1% change from baseline). Thereafter comparing the mean increases in HR from left with those from right sympathetic stimulation at successive levels, the response to left sympathetic stimulation at T4–T5 was 145.4 ± 3.9 bpm (15.1 ± 2.7%), and to right sympathetic stimulation 167.6 ± 11.5 bpm (25.9 ± 7.5%); T3–T4, left sympathetic stimulation increased HR to 155.0 ± 6.5 bpm (19.0 ± 4.4%), right sympathetic to 170.4 ± 7.0 bpm (29.0 ± 4.3%); T2–T3, left increased to 171.3 ± 5.3 bpm (29.4 ± 4.6%) and right to 189.5 ± 5.7 bpm (43.6 ± 4.7%); T1–T2, left increased to 181.8 ± 7.6 bpm (41.1 ± 5.6%) and right sympathetic to 211.8 ± 6.9 bpm (59.9 ± 6.0%) (Fig. 1a & b). At T5–T6 the LVP, measured during constant ventricular pacing, increased to 38.8 ± 6.1 mmHg (13.0 ± 2.1% from baseline) on stimulating the left sympathetic chain but there was no effect of stimulating the right (Fig. 1c & d). At each of the succeeding segments left sympathetic chain stimulation elicited larger increases in LVP compared to right stimulation as follows: T4–T5, left side effect was 40.5 ± 4.0 mmHg (17.4 ± 3.0%), right was 36.8 ± 4.0 mmHg (5.6 ± 1.5%); T3–T4, left was 43.6 ± 4.0 mmHg (20.9 ± 2.6%), right was 38.6 ± 4.0 mmHg (7.0 ± 1.6%); T2-T3 left was 45.3 ± 3.5 mmHg (28.8 ± 2.4%), right was 38.5 ± 3.4 mmHg (15.1 ± 2.3%); T1–T2 left was 51.3 ± 3.9 mmHg (40.3 ± 5.4%), right was 40.7 ± 3.7 mmHg (20.7 ± 3.2%). At all levels the left sympathetic chain was dominant and T1–T2 caused the largest increase.

### 1.2 Ventriculo-atrial (VA) conduction

**Fig. 2:**
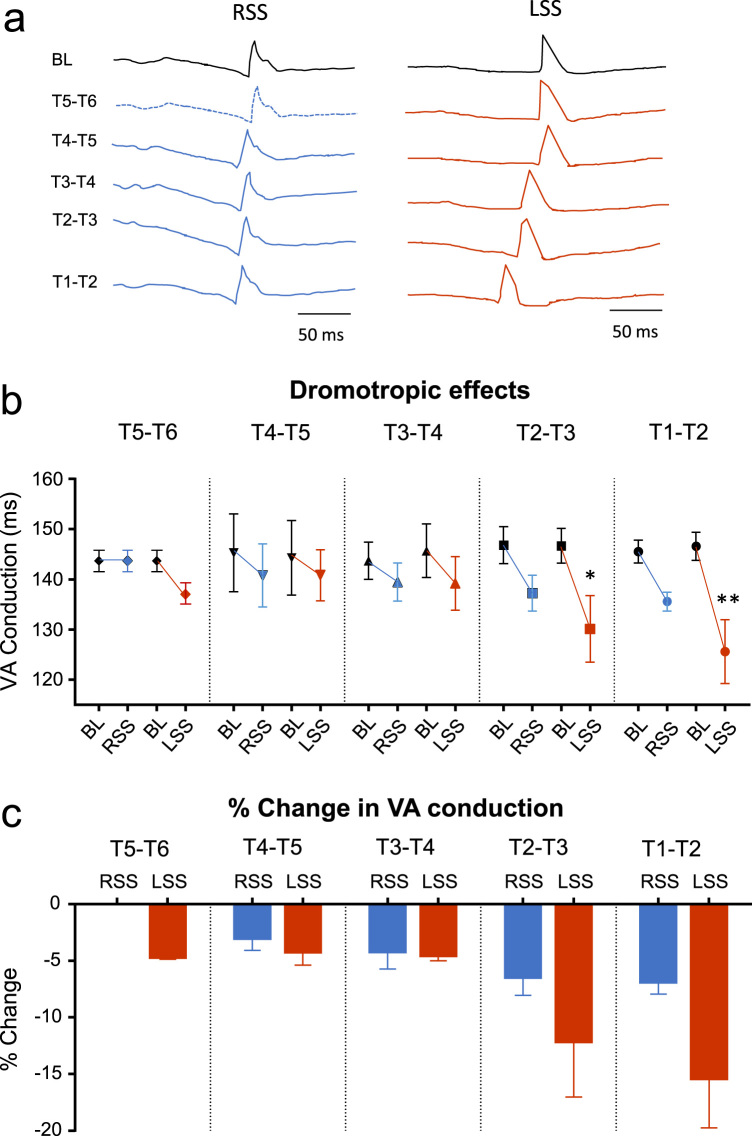
Dromotropic effects upon right and left sympathetic stimulation between T1–T6. (a) Raw data traces of the right atrial electrogram during baseline (BL) and right sympathetic stimulation (RSS) and left sympathetic stimulation (LSS) between T1–T6. (b) Dromotropic effects from BL to RSS and BL to LSS at spinal segments T1–T6. (c) Percentage change in ventriculo-atrial (VA) conduction for RSS and LSS at spinal segments T1–T6. Data represent mean ± SEM. *P< 0.05, **P< 0.01.

Dromotropic effects were measured during constant pacing with left and right sympathetic chain stimulation at each electrode pairing between T1 and T6 (Fig. 2a). A reduction in VA conduction was caused by both left and right sympathetic chain stimulation but the effects were largest following left stimulation. At T5–T6 left sympathetic stimulation decreased VA conduction from 143.7 ± 1.8 ms to 137 ± 1.7 ms (−4.6 ± 0.2%) as shown in Fig. 2b & c. There was no effect of right stimulation at this level. At T4–T5 and T3–T4 stimulation, both left and right sides elicited similar reductions to T5–T6 on the left side. More rostral the reductions were larger so at T2–T3 left side was 130.1 ± 6.7 ms (−12 ± 5.0%) and right was less at 137.2 ± 3.5 ms (−6.4 ± 1.7%). The reduction in VA conduction was largest at T1–T2 where left stimulation caused a change to 125.6 ± 6.6 ms (−15.5 ± 0.2%) whereas the right side was 135.6 ± 1.9 ms (−6.8 ± 1.1%).

### 1.3 Effective refractory period (ERP)

**Fig. 3:**
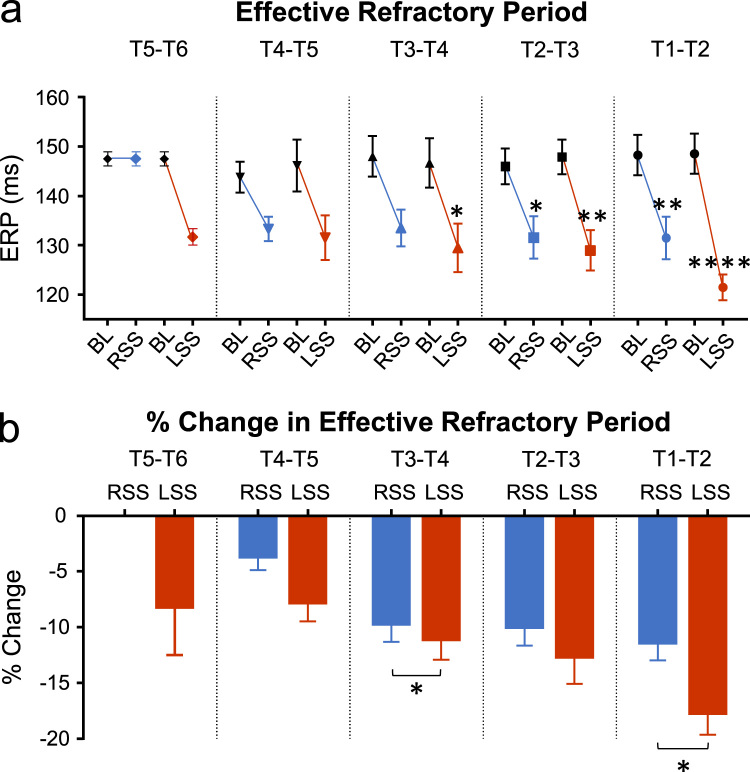
Changes in effective refractory period with right and left sympathetic stimulation at levels stimulated between T1–T6. (a) Effects on effective refractory period (ERP) from baseline (BL) to right sympathetic stimulation (RSS) and BL to left sympathetic stimulation (LSS) at spinal segments T1–T6. (b) Percentage ERP change for RSS and LSS at spinal segments T1–T6. Data represent mean ± SEM. *P< 0.05, **P< 0.01, ****P< 0.0001.

Left sympathetic stimulation at T5–T6 decreased the ERP from 147.5 ± 1.4 ms to 131.7 ± 1.7 ms, a change of –8.1 ± 4.4% (Fig. 3) but there was no effect of right sympathetic at this level. At subsequent levels the ERP was further shortened by stimulation of each sympathetic chain. At T4–T5 on the left the ERP reduction was −7.7 ± 1.8%; right at T4–T5 was 3.6 ± 1.3%; T3–T4 left was −11.0 ± 1.9%, right was −9.6 ± 1.7%; T2–T3 left was −12.6 ± 2.5%, right was −9.9 ± 1.8%. T1–T2 stimulation on the left side caused a much larger reduction in ERP to 121.5 ± 2.6 ms (−17.6 ± 2.0%). A similar enhancement was not observed for right side stimulation where the reduction was −11.3 ± 1.7%, not significantly different to the previous segment.

### 1.4 Electrical restitution of MAP duration

**Fig. 4:**
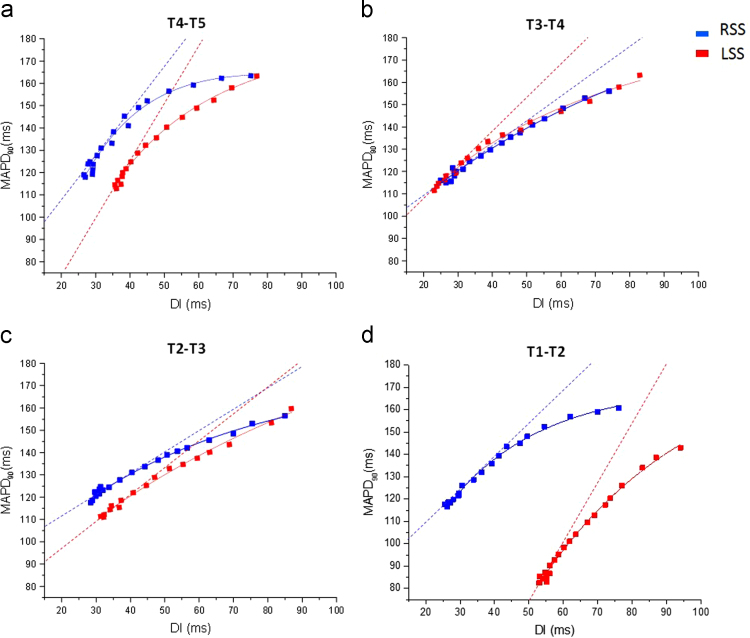
Monophasic action potential duration (MAPD) restitution slopes during segemental right and left sympathetic stimulation. Restitution (RT) slopes for right sympathetic stimulation (RSS) and left sympathetic stimulation at (a) T4–T5, (b) T3–T4, (c) T2–T3 and (d) T1–T2 with exponential curve fit (MAPD90 = maximum MAPD90 [1-e-DI/τ]). Dotted lines represent the maximum slope of restitution.

Restitution curves of the MAP duration at corresponding diastolic intervals were plotted, during baseline, left sympathetic stimulation and right sympathetic stimulation as shown in Fig. 4. The maximum slope of restitution was steepest during left sympathetic chain stimulation at T4–T5.

## 2 Experimental design, materials and methods

The left and right sympathetic chains were stimulated with a train of square wave pulses at 5–7 Hz via pairs of electrodes at five sites, T5–T6, T4–T5, T3–T4, T2–T3 and T1–T2 at x2 threshold, in the innervated isolated heart preparation from adult male New Zealand white rabbits (1.7–2.5 kg, *n* = 18). This preparation has been described previously [2], [3]. Sinus rate, left ventricular pressure, retrograde ventriculo-atrial conduction, monophasic action potential duration, effective refractory period and electrical restitution were measured.

All procedures conformed to the ethical guidelines in the Animal Scientific Procedure Act 1986 (ASPA), in accordance with Guide for the Care and Use of Laboratory Animals published by the US National Institute of Health (NIH Publication No. 85-23, revised 1985), and followed the criteria of the EU legislation on the protection of animals used for scientific purposes (Directive 2010/63/EU, 2010).

A fluid filled latex balloon was inserted into the left ventricle to measure the left ventricular pressure (LVP) via a pressure transducer (MLT0380/D ADInstruments Ltd, Charlgrove, UK). Heart rate was calculated using cyclic measurements from the LVP. Platinum hook electrodes (Grass Instruments, Astro-Med Inc., USA) were attached to the right atria in order the measure the atrial electrogram. Dromotropic effects of left and right sympathetic stimulation at different levels were measured from right atrial electrograms during constant ventricular electrical pacing to obtain VA conduction by measuring the delay from right ventricle pacing spike to the atrial electrogram.

Monophasic action potentials (MAPs) were recorded extracellularly from the left ventricular epicardial surface of the heart at the base and apex, by applying two MAP electrodes (Harvard Apparatus Ltd, Holliston, Massachusetts, US. Model number 73–0150), gently onto the surface. A pacing catheter (ADinstruments Ltd, Chalgrove, UK) was inserted into the right ventricle to deliver a stimulus to the heart at double the diastolic pacing threshold using a constant current stimulator. The restitution (RT) protocol was applied as described previously [4] until the effective refractory period (ERP) was reached. The ERP was defined as the longest S1-S2 interval that failed to capture the S2 beat. MAP duration restitution curves were constructed by plotting S2 MAP duration vs. diastolic interval (DI = interval between the S1-and S2-MAP signals minus S1-MAPD90). MAP duration was identified from time of activation (Tact) to 90% of repolarization (MAPD90) using the programme NewMap (Francis Burton, Glasgow University, UK). Using Microcal Origin (v 6.0, Origin, San Diego, CA, US), an exponential curve was formulated using the following function: MAPD90 = maximum MAPD90[1-e-DI/t] where t = time constant and the maximum slope of the curve (RT slope) was acquired by measuring the peak value of the first derivative.

The Powerlab 16/30 system (AD Instruments Ltd, Chalgrove, UK) was used to record the signals, which were processed at 2 kHz. Data are mean ± SEM; compared using ANOVA or paired *t*-test for which statistical significance was taken at 5% level (*p*< 0.05). Discussion of the data can be found in Chauhan et al. [1].
